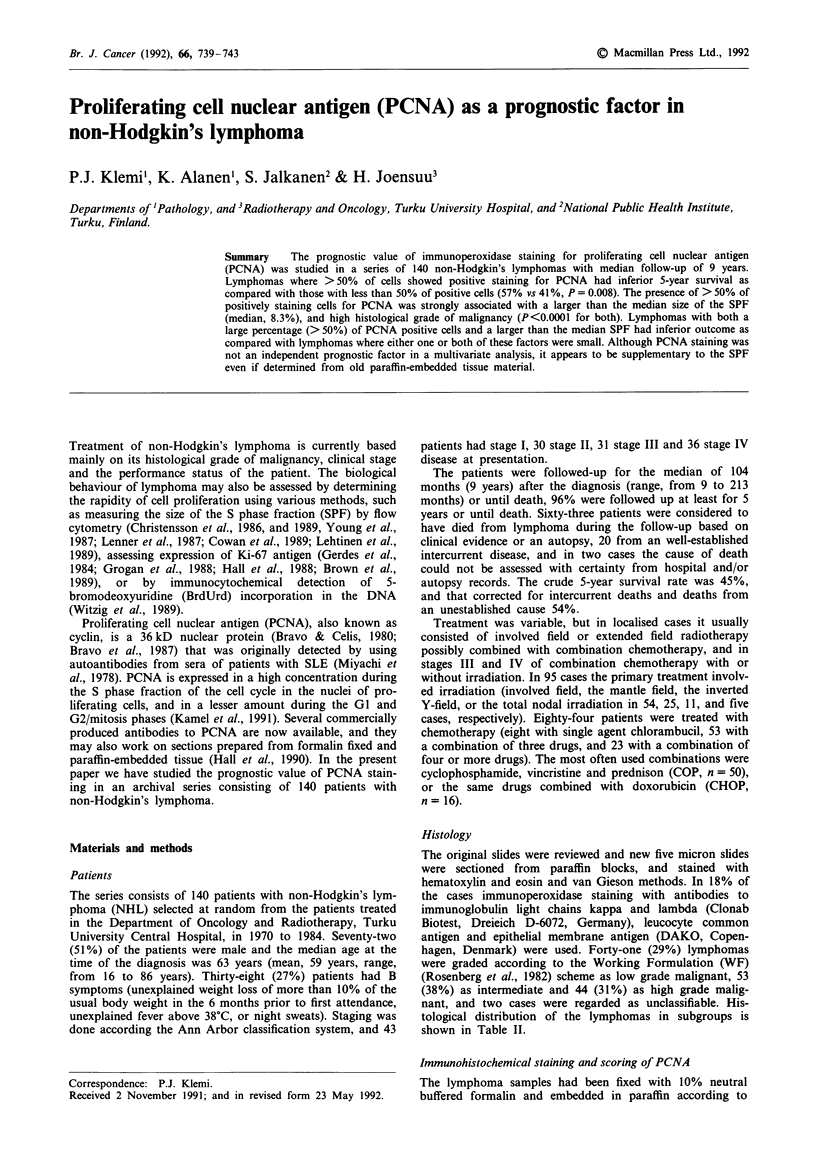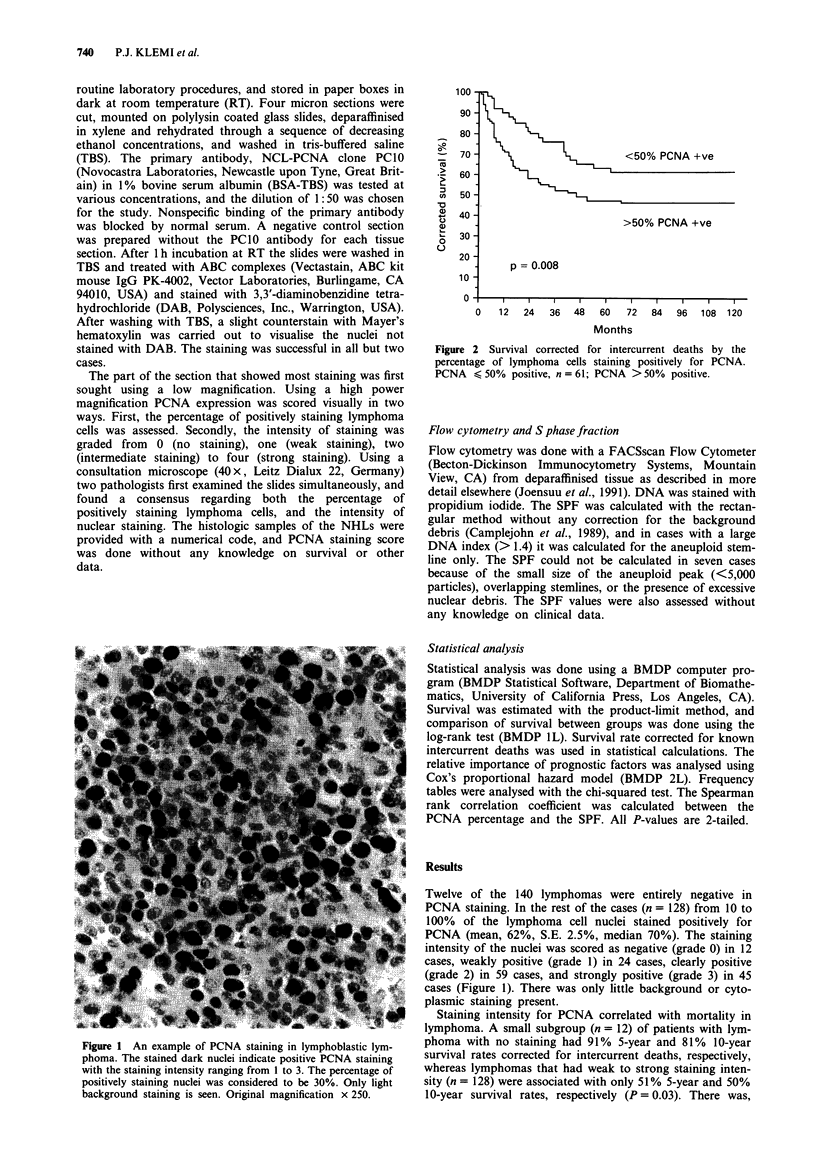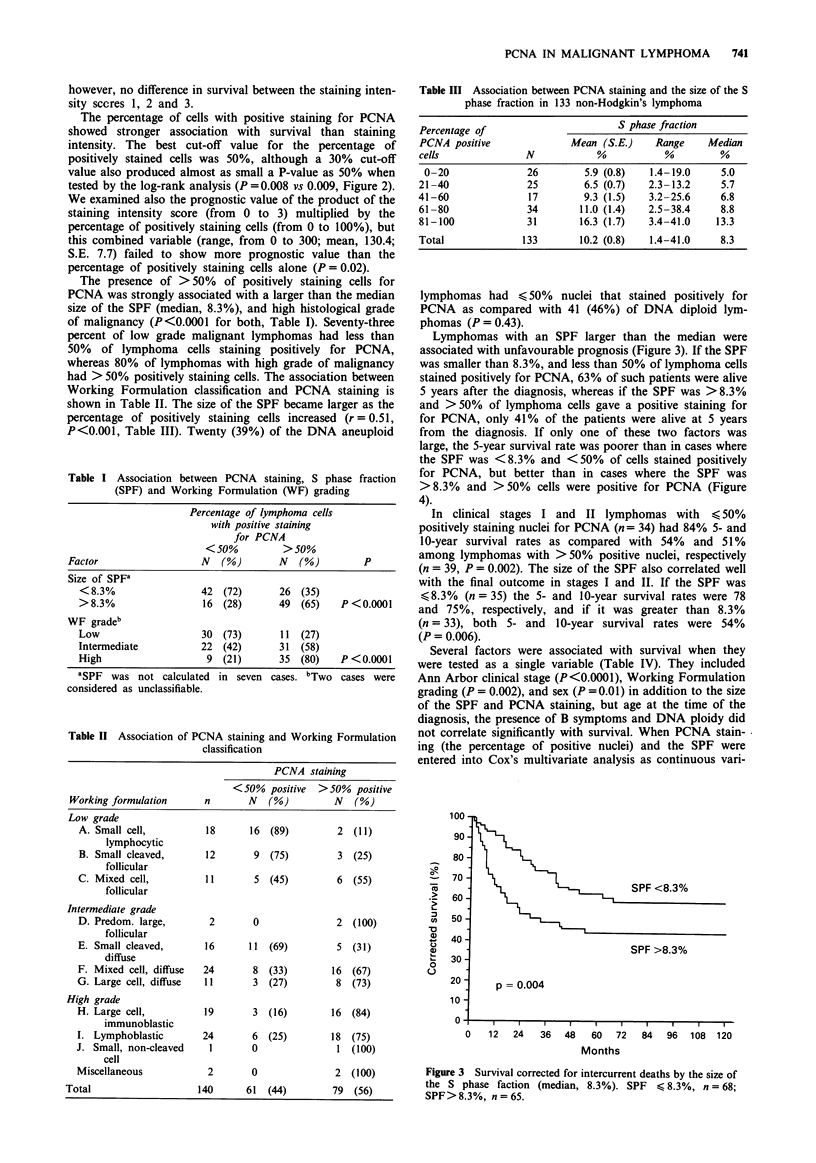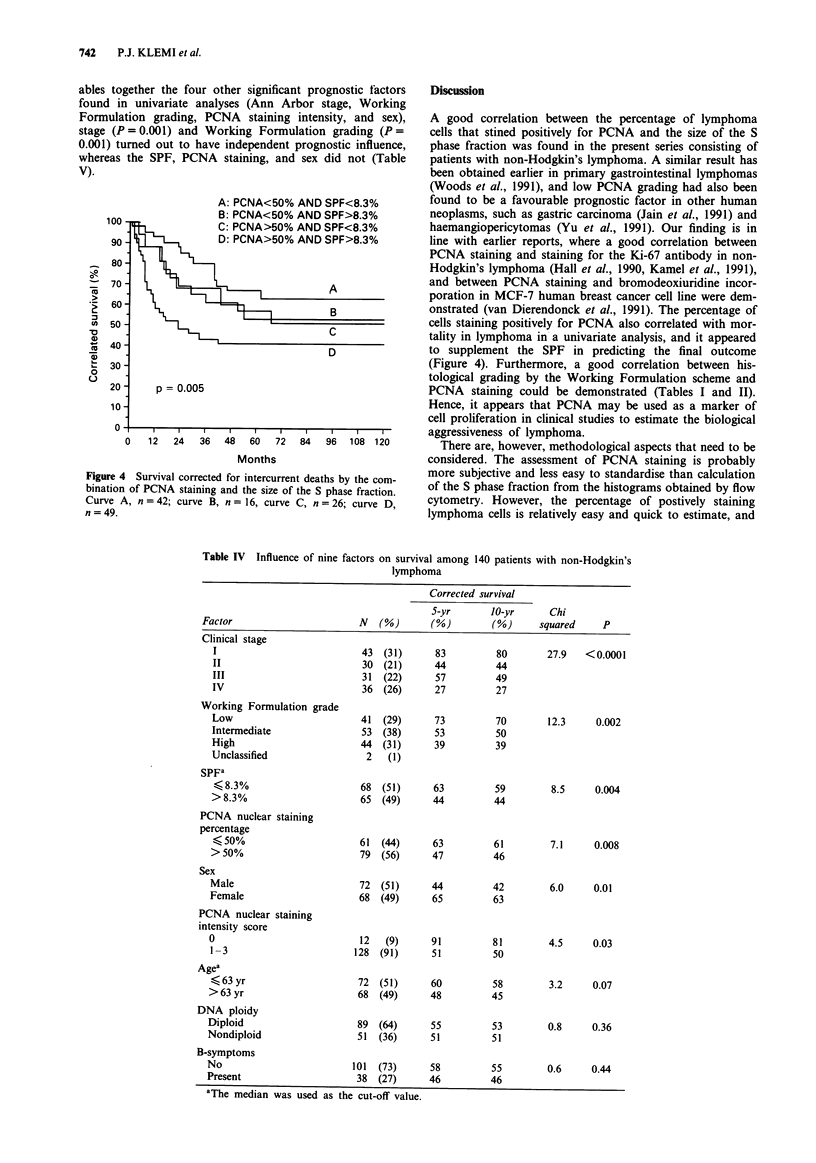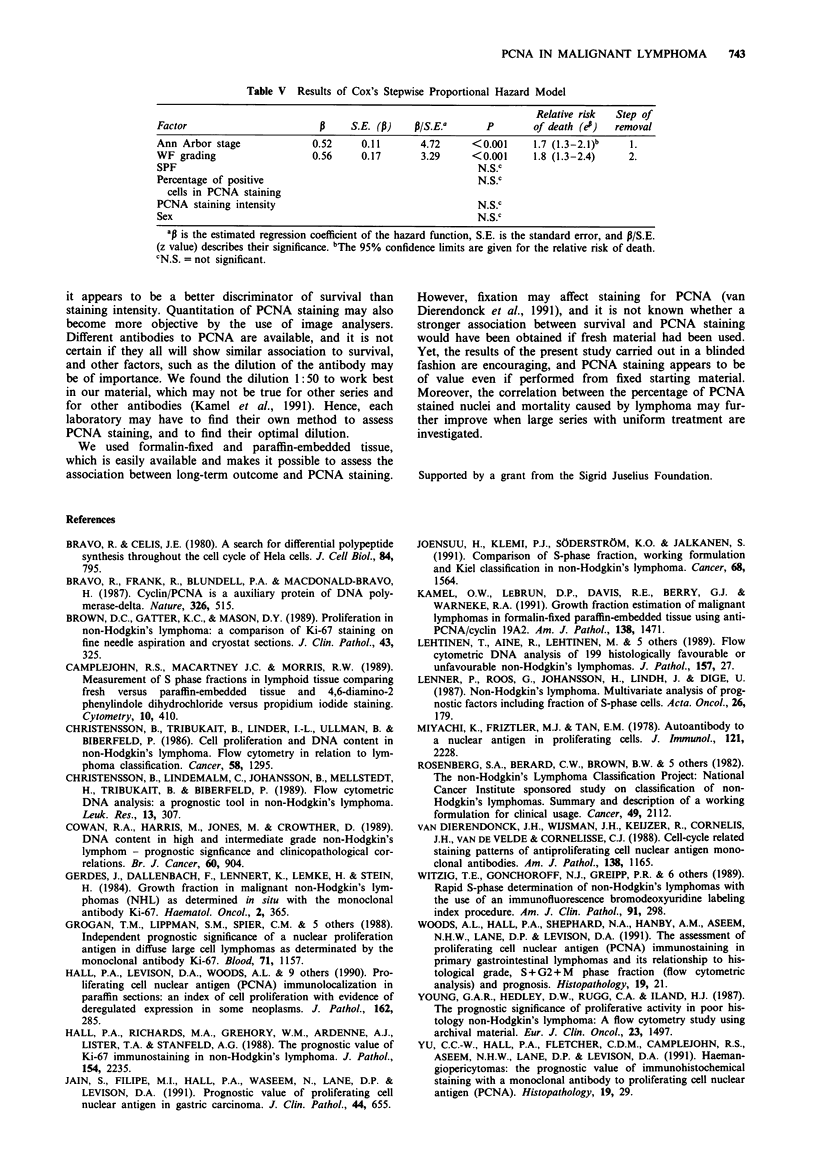# Proliferating cell nuclear antigen (PCNA) as a prognostic factor in non-Hodgkin's lymphoma.

**DOI:** 10.1038/bjc.1992.349

**Published:** 1992-10

**Authors:** P. J. Klemi, K. Alanen, S. Jalkanen, H. Joensuu

**Affiliations:** Department of Pathology, Turku University Hospital, Finland.

## Abstract

**Images:**


Br. J. Cancer (1992), 66, 739-743 © Macmillan Press Ltd., 1992

### Proliferating cell nuclear antigen (PCNA) as a prognostic factor in non-Hodgkin's lymphoma

P.J. Klemi[1], K. Alanen[1], S. Jalkanen[2] & H. Joensuu[3]

*Departments of [1]Pathology, and [3]Radiotherapy and Oncology, Turku University Hospital, and [2]National Public Health Institute, Turku, Finland.*

**Summary** The prognostic value of immunoperoxidase staining for proliferating cell nuclear antigen (PCNA) was studied in a series of 140 non-Hodgkin's lymphomas with median follow-up of 9 years. Lymphomas where >50% of cells showed positive staining for PCNA had inferior 5-year survival as compared with those with less than 50% of positive cells (57% *vs* 41%, $P = 0.008$). The presence of >50% of positively staining cells for PCNA was strongly associated with a larger than the median size of the SPF (median, 8.3%), and high histological grade of malignancy ($P < 0.0001$ for both). Lymphomas with both a large percentage (>50%) of PCNA positive cells and a larger than the median SPF had inferior outcome as compared with lymphomas where either one or both of these factors were small. Although PCNA staining was not an independent prognostic factor in a multivariate analysis, it appears to be supplementary to the SPF even if determined from old paraffin-embedded tissue material.

Treatment of non-Hodgkin's lymphoma is currently based mainly on its histological grade of malignancy, clinical stage and the performance status of the patient. The biological behaviour of lymphoma may also be assessed by determining the rapidity of cell proliferation using various methods, such as measuring the size of the S phase fraction (SPF) by flow cytometry (Christensson *et al.*, 1986, and 1989, Young *et al.*, 1987; Lenner *et al.*, 1987; Cowan *et al.*, 1989; Lehtinen *et al.*, 1989), assessing expression of Ki-67 antigen (Gerdes *et al.*, 1984; Grogan *et al.*, 1988; Hall *et al.*, 1988; Brown *et al.*, 1989), or by immunocytochemical detection of 5-bromodeoxyuridine (BrdUrd) incorporation in the DNA (Witzig *et al.*, 1989).

Proliferating cell nuclear antigen (PCNA), also known as cyclin, is a 36 kD nuclear protein (Bravo & Celis, 1980; Bravo *et al.*, 1987) that was originally detected by using autoantibodies from sera of patients with SLE (Miyachi *et al.*, 1978). PCNA is expressed in a high concentration during the S phase fraction of the cell cycle in the nuclei of proliferating cells, and in a lesser amount during the G1 and G2/mitosis phases (Kamel *et al.*, 1991). Several commercially produced antibodies to PCNA are now available, and they may also work on sections prepared from formalin fixed and paraffin-embedded tissue (Hall *et al.*, 1990). In the present paper we have studied the prognostic value of PCNA staining in an archival series consisting of 140 patients with non-Hodgkin's lymphoma.

### Materials and methods

### *Patients*

The series consists of 140 patients with non-Hodgkin's lymphoma (NHL) selected at random from the patients treated in the Department of Oncology and Radiotherapy, Turku University Central Hospital, in 1970 to 1984. Seventy-two (51%) of the patients were male and the median age at the time of the diagnosis was 63 years (mean, 59 years, range, from 16 to 86 years). Thirty-eight (27%) patients had B symptoms (unexplained weight loss of more than 10% of the usual body weight in the 6 months prior to first attendance, unexplained fever above 38°C, or night sweats). Staging was done according the Ann Arbor classification system, and 43 patients had stage I, 30 stage II, 31 stage III and 36 stage IV disease at presentation.

The patients were followed-up for the median of 104 months (9 years) after the diagnosis (range, from 9 to 213 months) or until death, 96% were followed up at least for 5 years or until death. Sixty-three patients were considered to have died from lymphoma during the follow-up based on clinical evidence or an autopsy, 20 from an well-established intercurrent disease, and in two cases the cause of death could not be assessed with certainty from hospital and/or autopsy records. The crude 5-year survival rate was 45%, and that corrected for intercurrent deaths and deaths from an unestablished cause 54%.

Treatment was variable, but in localised cases it usually consisted of involved field or extended field radiotherapy possibly combined with combination chemotherapy, and in stages III and IV of combination chemotherapy with or without irradiation. In 95 cases the primary treatment involved irradiation (involved field, the mantle field, the inverted Y-field, or the total nodal irradiation in 54, 25, 11, and five cases, respectively). Eighty-four patients were treated with chemotherapy (eight with single agent chloramhucil, 53 with a combination of three drugs, and 23 with a combination of four or more drugs). The most often used combinations were cyclophosphamide, vincristine and prednison (COP, $n = 50$), or the same drugs combined with doxorubicin (CHOP, $n = 16$).

### *Histology*

The original slides were reviewed and new five micron slides were sectioned from paraffin blocks, and stained with hematoxylin and eosin and van Gieson methods. In 18% of the cases immunoperoxidase staining with antibodies to immunoglobulin light chains kappa and lambda (Clonab Biotest, Dreieich D-6072, Germany), leucocyte common antigen and epithelial membrane antigen (DAKO, Copenhagen, Denmark) were used. Forty-one (29%) lymphomas were graded according to the Working Formulation (WF) (Rosenberg *et al.*, 1982) scheme as low grade malignant, 53 (38%) as intermediate and 44 (31%) as high grade malignant, and two cases were regarded as unclassifiable. Histological distribution of the lymphomas in subgroups is shown in Table II.

### *Immunohistochemical staining and scoring of PCNA*

The lymphoma samples had been fixed with 10% neutral buffered formalin and embedded in paraffin according to

Correspondence: P.J. Klemi.
Received 2 November 1991; and in revised form 23 May 1992.

routine laboratory procedures, and stored in paper boxes in dark at room temperature (RT). Four micron sections were cut, mounted on polylysin coated glass slides, deparaffinised in xylene and rehydrated through a sequence of decreasing ethanol concentrations, and washed in tris-buffered saline (TBS). The primary antibody, NCL-PCNA clone PC10 (Novocastra Laboratories, Newcastle upon Tyne, Great Britain) in 1% bovine serum albumin (BSA-TBS) was tested at various concentrations, and the dilution of 1:50 was chosen for the study. Nonspecific binding of the primary antibody was blocked by normal serum. A negative control section was prepared without the PC10 antibody for each tissue section. After 1 h incubation at RT the slides were washed in TBS and treated with ABC complexes (Vectastain, ABC kit mouse IgG PK-4002, Vector Laboratories, Burlingame, CA 94010, USA) and stained with 3,3'-diaminobenzidine tetrahydrochloride (DAB, Polysciences, Inc., Warrington, USA). After washing with TBS, a slight counterstain with Mayer's hematoxylin was carried out to visualise the nuclei not stained with DAB. The staining was successful in all but two cases.

The part of the section that showed most staining was first sought using a low magnification. Using a high power magnification PCNA expression was scored visually in two ways. First, the percentage of positively staining lymphoma cells was assessed. Secondly, the intensity of staining was graded from 0 (no staining), one (weak staining), two (intermediate staining) to four (strong staining). Using a consultation microscope (40×, Leitz Dialux 22, Germany) two pathologists first examined the slides simultaneously, and found a consensus regarding both the percentage of positively staining lymphoma cells, and the intensity of nuclear staining. The histologic samples of the NHLs were provided with a numerical code, and PCNA staining score was done without any knowledge on survival or other data.

**Figure 2** Survival corrected for intercurrent deaths by the percentage of lymphoma cells staining positively for PCNA. PCNA ⩽50% positive, $n = 61$; PCNA >50% positive.

*Flow cytometry and S phase fraction*

Flow cytometry was done with a FACSscan Flow Cytometer (Becton-Dickinson Immunocytometry Systems, Mountain View, CA) from deparaffinised tissue as described in more detail elsewhere (Joensuu *et al*., 1991). DNA was stained with propidium iodide. The SPF was calculated with the rectangular method without any correction for the background debris (Camplejohn *et al*., 1989), and in cases with a large DNA index (>1.4) it was calculated for the aneuploid stemline only. The SPF could not be calculated in seven cases because of the small size of the aneuploid peak (<5,000 particles), overlapping stemlines, or the presence of excessive nuclear debris. The SPF values were also assessed without any knowledge on clinical data.

**Figure 1** An example of PCNA staining in lymphoblastic lymphoma. The stained dark nuclei indicate positive PCNA staining with the staining intensity ranging from 1 to 3. The percentage of positively staining nuclei was considered to be 30%. Only light background staining is seen. Original magnification ×250.

*Statistical analysis*

Statistical analysis was done using a BMDP computer program (BMDP Statistical Software, Department of Biomathematics, University of California Press, Los Angeles, CA). Survival was estimated with the product-limit method, and comparison of survival between groups was done using the log-rank test (BMDP 1L). Survival rate corrected for known intercurrent deaths was used in statistical calculations. The relative importance of prognostic factors was analysed using Cox's proportional hazard model (BMDP 2L). Frequency tables were analysed with the chi-squared test. The Spearman rank correlation coefficient was calculated between the PCNA percentage and the SPF. All *P*-values are 2-tailed.

### Results

Twelve of the 140 lymphomas were entirely negative in PCNA staining. In the rest of the cases ($n = 128$) from 10 to 100% of the lymphoma cell nuclei stained positively for PCNA (mean, 62%, S.E. 2.5%, median 70%). The staining intensity of the nuclei was scored as negative (grade 0) in 12 cases, weakly positive (grade 1) in 24 cases, clearly positive (grade 2) in 59 cases, and strongly positive (grade 3) in 45 cases (Figure 1). There was only little background or cytoplasmic staining present.

Staining intensity for PCNA correlated with mortality in lymphoma. A small subgroup ($n = 12$) of patients with lymphoma with no staining had 91% 5-year and 81% 10-year survival rates corrected for intercurrent deaths, respectively, whereas lymphomas that had weak to strong staining intensity ($n = 128$) were associated with only 51% 5-year and 50% 10-year survival rates, respectively ($P = 0.03$). There was, however, no difference in survival between the staining intensity scores 1, 2 and 3.

The percentage of cells with positive staining for PCNA showed stronger association with survival than staining intensity. The best cut-off value for the percentage of positively stained cells was 50%, although a 30% cut-off value also produced almost as small a P-value as 50% when tested by the log-rank analysis ($P = 0.008$ *vs* 0.009, Figure 2). We examined also the prognostic value of the product of the staining intensity score (from 0 to 3) multiplied by the percentage of positively staining cells (from 0 to 100%), but this combined variable (range, from 0 to 300; mean, 130.4; S.E. 7.7) failed to show more prognostic value than the percentage of positively staining cells alone ($P = 0.02$).

The presence of >50% of positively staining cells for PCNA was strongly associated with a larger than the median size of the SPF (median, 8.3%), and high histological grade of malignancy ($P < 0.0001$ for both, Table I). Seventy-three percent of low grade malignant lymphomas had less than 50% of lymphoma cells staining positively for PCNA, whereas 80% of lymphomas with high grade of malignancy had >50% positively staining cells. The association between Working Formulation classification and PCNA staining is shown in Table II. The size of the SPF became larger as the percentage of positively staining cells increased ($r = 0.51$, $P < 0.001$, Table III). Twenty (39%) of the DNA aneuploid lymphomas had ⩽50% nuclei that stained positively for PCNA as compared with 41 (46%) of DNA diploid lymphomas ($P = 0.43$).

Lymphomas with an SPF larger than the median were associated with unfavourable prognosis (Figure 3). If the SPF was smaller than 8.3%, and less than 50% of lymphoma cells stained positively for PCNA, 63% of such patients were alive 5 years after the diagnosis, whereas if the SPF was >8.3% and >50% of lymphoma cells gave a positive staining for for PCNA, only 41% of the patients were alive at 5 years from the diagnosis. If only one of these two factors was large, the 5-year survival rate was poorer than in cases where the SPF was <8.3% and <50% of cells stained positively for PCNA, but better than in cases where the SPF was >8.3% and >50% cells were positive for PCNA (Figure 4).

In clinical stages I and II lymphomas with ⩽50% positively staining nuclei for PCNA ($n = 34$) had 84% 5- and 10-year survival rates as compared with 54% and 51% among lymphomas with >50% positive nuclei, respectively ($n = 39$, $P = 0.002$). The size of the SPF also correlated well with the final outcome in stages I and II. If the SPF was ⩽8.3% ($n = 35$) the 5- and 10-year survival rates were 78 and 75%, respectively, and if it was greater than 8.3% ($n = 33$), both 5- and 10-year survival rates were 54% ($P = 0.006$).

Several factors were associated with survival when they were tested as a single variable (Table IV). They included Ann Arbor clinical stage ($P < 0.0001$), Working Formulation grading ($P = 0.002$), and sex ($P = 0.01$) in addition to the size of the SPF and PCNA staining, but age at the time of the diagnosis, the presence of B symptoms and DNA ploidy did not correlate significantly with survival. When PCNA staining (the percentage of positive nuclei) and the SPF were entered into Cox's multivariate analysis as continuous vari-

**Table I** Association between PCNA staining, S phase fraction (SPF) and Working Formulation (WF) grading

| Factor | Percentage of lymphoma cells with positive staining for PCNA <50% N (%) | >50% N (%) | P |
|---|---|---|---|
| Size of SPF[a] | | | |
| <8.3% | 42 (72) | 26 (35) | |
| >8.3% | 16 (28) | 49 (65) | P<0.0001 |
| WF grade[b] | | | |
| Low | 30 (73) | 11 (27) | |
| Intermediate | 22 (42) | 31 (58) | |
| High | 9 (21) | 35 (80) | P<0.0001 |

[a]SPF was not calculated in seven cases. [b]Two cases were considered as unclassifiable.

**Table II** Association of PCNA staining and Working Formulation classification

| Working formulation | n | PCNA staining <50% positive N (%) | >50% positive N (%) |
|---|---|---|---|
| *Low grade* | | | |
| A. Small cell, lymphocytic | 18 | 16 (89) | 2 (11) |
| B. Small cleaved, follicular | 12 | 9 (75) | 3 (25) |
| C. Mixed cell, follicular | 11 | 5 (45) | 6 (55) |
| *Intermediate grade* | | | |
| D. Predom. large, follicular | 2 | 0 | 2 (100) |
| E. Small cleaved, diffuse | 16 | 11 (69) | 5 (31) |
| F. Mixed cell, diffuse | 24 | 8 (33) | 16 (67) |
| G. Large cell, diffuse | 11 | 3 (27) | 8 (73) |
| *High grade* | | | |
| H. Large cell, immunoblastic | 19 | 3 (16) | 16 (84) |
| I. Lymphoblastic | 24 | 6 (25) | 18 (75) |
| J. Small, non-cleaved cell | 1 | 0 | 1 (100) |
| Miscellaneous | 2 | 0 | 2 (100) |
| Total | 140 | 61 (44) | 79 (56) |

**Table III** Association between PCNA staining and the size of the S phase fraction in 133 non-Hodgkin's lymphoma

| Percentage of PCNA positive cells | N | S phase fraction Mean (S.E.) % | Range % | Median % |
|---|---|---|---|---|
| 0-20 | 26 | 5.9 (0.8) | 1.4-19.0 | 5.0 |
| 21-40 | 25 | 6.5 (0.7) | 2.3-13.2 | 5.7 |
| 41-60 | 17 | 9.3 (1.5) | 3.2-25.6 | 6.8 |
| 61-80 | 34 | 11.0 (1.4) | 2.5-38.4 | 8.8 |
| 81-100 | 31 | 16.3 (1.7) | 3.4-41.0 | 13.3 |
| Total | 133 | 10.2 (0.8) | 1.4-41.0 | 8.3 |

**Figure 3** Survival corrected for intercurrent deaths by the size of the S phase faction (median, 8.3%). SPF ⩽8.3%, $n = 68$; SPF>8.3%, $n = 65$.

ables together the four other significant prognostic factors found in univariate analyses (Ann Arbor stage, Working Formulation grading, PCNA staining intensity, and sex), stage ($P = 0.001$) and Working Formulation grading ($P = 0.001$) turned out to have independent prognostic influence, whereas the SPF, PCNA staining, and sex did not (Table V).

**Figure 4** Survival corrected for intercurrent deaths by the combination of PCNA staining and the size of the S phase fraction. Curve A, $n = 42$; curve B, $n = 16$, curve C, $n = 26$; curve D, $n = 49$.

### Discussion

A good correlation between the percentage of lymphoma cells that stined positively for PCNA and the size of the S phase fraction was found in the present series consisting of patients with non-Hodgkin's lymphoma. A similar result has been obtained earlier in primary gastrointestinal lymphomas (Woods *et al.*, 1991), and low PCNA grading had also been found to be a favourable prognostic factor in other human neoplasms, such as gastric carcinoma (Jain *et al.*, 1991) and haemangiopericytomas (Yu *et al.*, 1991). Our finding is in line with earlier reports, where a good correlation between PCNA staining and staining for the Ki-67 antibody in non-Hodgkin's lymphoma (Hall *et al.*, 1990, Kamel *et al.*, 1991), and between PCNA staining and bromodeoxiuridine incorporation in MCF-7 human breast cancer cell line were demonstrated (van Dierendonck *et al.*, 1991). The percentage of cells staining positively for PCNA also correlated with mortality in lymphoma in a univariate analysis, and it appeared to supplement the SPF in predicting the final outcome (Figure 4). Furthermore, a good correlation between histological grading by the Working Formulation scheme and PCNA staining could be demonstrated (Tables I and II). Hence, it appears that PCNA may be used as a marker of cell proliferation in clinical studies to estimate the biological aggressiveness of lymphoma.

There are, however, methodological aspects that need to be considered. The assessment of PCNA staining is probably more subjective and less easy to standardise than calculation of the S phase fraction from the histograms obtained by flow cytometry. However, the percentage of postively staining lymphoma cells is relatively easy and quick to estimate, and

**Table IV** Influence of nine factors on survival among 140 patients with non-Hodgkin's lymphoma

| Factor | N | (%) | Corrected survival 5-yr (%) | Corrected survival 10-yr (%) | Chi squared | P |
|---|---|---|---|---|---|---|
| Clinical stage | | | | | | |
| I | 43 | (31) | 83 | 80 | 27.9 | <0.0001 |
| II | 30 | (21) | 44 | 44 | | |
| III | 31 | (22) | 57 | 49 | | |
| IV | 36 | (26) | 27 | 27 | | |
| Working Formulation grade | | | | | | |
| Low | 41 | (29) | 73 | 70 | 12.3 | 0.002 |
| Intermediate | 53 | (38) | 53 | 50 | | |
| High | 44 | (31) | 39 | 39 | | |
| Unclassified | 2 | (1) | | | | |
| SPF[a] | | | | | | |
| $\leqslant$8.3% | 68 | (51) | 63 | 59 | 8.5 | 0.004 |
| >8.3% | 65 | (49) | 44 | 44 | | |
| PCNA nuclear staining percentage | | | | | | |
| $\leqslant$50% | 61 | (44) | 63 | 61 | 7.1 | 0.008 |
| >50% | 79 | (56) | 47 | 46 | | |
| Sex | | | | | | |
| Male | 72 | (51) | 44 | 42 | 6.0 | 0.01 |
| Female | 68 | (49) | 65 | 63 | | |
| PCNA nuclear staining intensity score | | | | | | |
| 0 | 12 | (9) | 91 | 81 | 4.5 | 0.03 |
| 1–3 | 128 | (91) | 51 | 50 | | |
| Age[a] | | | | | | |
| $\leqslant$63 yr | 72 | (51) | 60 | 58 | 3.2 | 0.07 |
| >63 yr | 68 | (49) | 48 | 45 | | |
| DNA ploidy | | | | | | |
| Diploid | 89 | (64) | 55 | 53 | 0.8 | 0.36 |
| Nondiploid | 51 | (36) | 51 | 51 | | |
| B-symptoms | | | | | | |
| No | 101 | (73) | 58 | 55 | 0.6 | 0.44 |
| Present | 38 | (27) | 46 | 46 | | |

[a]The median was used as the cut-off value.

**Table V** Results of Cox's Stepwise Proportional Hazard Model

| *Factor* | $\beta$ | *S.E.* ($\beta$) | $\beta$/*S.E.*[a] | *P* | *Relative risk of death* ($e^{\beta}$) | *Step of removal* |
|---|---|---|---|---|---|---|
| Ann Arbor stage | 0.52 | 0.11 | 4.72 | <0.001 | 1.7 (1.3–2.1)[b] | 1. |
| WF grading | 0.56 | 0.17 | 3.29 | <0.001 | 1.8 (1.3–2.4) | 2. |
| SPF | | | | N.S.[c] | | |
| Percentage of positive cells in PCNA staining | | | | N.S.[c] | | |
| PCNA staining intensity | | | | N.S.[c] | | |
| Sex | | | | N.S.[c] | | |

[a]$\beta$ is the estimated regression coefficient of the hazard function, S.E. is the standard error, and $\beta$/S.E. (z value) describes their significance. [b]The 95% confidence limits are given for the relative risk of death. [c]N.S. = not significant.

it appears to be a better discriminator of survival than staining intensity. Quantitation of PCNA staining may also become more objective by the use of image analysers. Different antibodies to PCNA are available, and it is not certain if they all will show similar association to survival, and other factors, such as the dilution of the antibody may be of importance. We found the dilution 1:50 to work best in our material, which may not be true for other series and for other antibodies (Kamel *et al.*, 1991). Hence, each laboratory may have to find their own method to assess PCNA staining, and to find their optimal dilution.

We used formalin-fixed and paraffin-embedded tissue, which is easily available and makes it possible to assess the association between long-term outcome and PCNA staining. However, fixation may affect staining for PCNA (van Dierendonck *et al.*, 1991), and it is not known whether a stronger association between survival and PCNA staining would have been obtained if fresh material had been used. Yet, the results of the present study carried out in a blinded fashion are encouraging, and PCNA staining appears to be of value even if performed from fixed starting material. Moreover, the correlation between the percentage of PCNA stained nuclei and mortality caused by lymphoma may further improve when large series with uniform treatment are investigated.

Supported by a grant from the Sigrid Juselius Foundation.

### References

BRAVO, R. & CELIS, J.E. (1980). A search for differential polypeptide synthesis throughout the cell cycle of Hela cells. *J. Cell Biol.*, **84**, 795.

BRAVO, R., FRANK, R., BLUNDELL, P.A. & MACDONALD-BRAVO, H. (1987). Cyclin/PCNA is a auxiliary protein of DNA polymerase-delta. *Nature*, **326**, 515.

BROWN, D.C., GATTER, K.C., & MASON, D.Y. (1989). Proliferation in non-Hodgkin's lymphoma: a comparison of Ki-67 staining on fine needle aspiration and cryostat sections. *J. Clin. Pathol.*, **43**, 325.

CAMPLEJOHN, R.S., MACARTNEY J.C. & MORRIS, R.W. (1989). Measurement of S phase fractions in lymphoid tissue comparing fresh versus paraffin-embedded tissue and 4,6-diamino-2 phenylindole dihydrochloride versus propidium iodide staining. *Cytometry*, **10**, 410.

CHRISTENSSON, B., TRIBUKAIT, B., LINDER, I.-L., ULLMAN, B. & BIBERFELD, P. (1986). Cell proliferation and DNA content in non-Hodgkin's lymphoma. Flow cytometry in relation to lymphoma classification. *Cancer*, **58**, 1295.

CHRISTENSSON, B., LINDEMALM, C., JOHANSSON, B., MELLSTEDT, H., TRIBUKAIT, B. & BIBERFELD, P. (1989). Flow cytometric DNA analysis: a prognostic tool in non-Hodgkin's lymphoma. *Leuk. Res.*, **13**, 307.

COWAN, R.A., HARRIS, M., JONES, M. & CROWTHER, D. (1989). DNA content in high and intermediate grade non-Hodgkin's lymphom – prognostic signficance and clinicopathological correlations. *Br. J. Cancer*, **60**, 904.

GERDES, J., DALLENBACH, F., LENNERT, K., LEMKE, H. & STEIN, H. (1984). Growth fraction in malignant non-Hodgkin's lymphomas (NHL) as determined *in situ* with the monoclonal antibody Ki-67. *Haematol. Oncol.*, **2**, 365.

GROGAN, T.M., LIPPMAN, S.M., SPIER, C.M. & 5 others (1988). Independent prognostic significance of a nuclear proliferation antigen in diffuse large cell lymphomas as determinated by the monoclonal antibody Ki-67. *Blood*, **71**, 1157.

HALL, P.A., LEVISON, D.A., WOODS, A.L. & 9 others (1990). Proliferating cell nuclear antigen (PCNA) immunolocalization in paraffin sections: an index of cell proliferation with evidence of deregulated expression in some neoplasms. *J. Pathol.*, **162**, 285.

HALL, P.A., RICHARDS, M.A., GREHORY, W.M., ARDENNE, A.J., LISTER, T.A. & STANFELD, A.G. (1988). The prognostic value of Ki-67 immunostaining in non-Hodgkin's lymphoma. *J. Pathol.*, **154**, 2235.

JAIN, S., FILIPE, M.I., HALL, P.A., WASEEM, N., LANE, D.P. & LEVISON, D.A. (1991). Prognostic value of proliferating cell nuclear antigen in gastric carcinoma. *J. Clin. Pathol.*, **44**, 655.

JOENSUU, H., KLEMI, P.J., SÖDERSTRÖM, K.O. & JALKANEN, S. (1991). Comparison of S-phase fraction, working formulation and Kiel classification in non-Hodgkin's lymphoma. *Cancer*, **68**, 1564.

KAMEL, O.W., LEBRUN, D.P., DAVIS, R.E., BERRY, G.J. & WARNEKE, R.A. (1991). Growth fraction estimation of malignant lymphomas in formalin-fixed paraffin-embedded tissue using anti-PCNA/cyclin 19A2. *Am. J. Pathol.*, **138**, 1471.

LEHTINEN, T., AINE, R., LEHTINEN, M. & 5 others (1989). Flow cytometric DNA analysis of 199 histologically favourable or unfavourable non-Hodgkin's lymphomas. *J. Pathol.*, **157**, 27.

LENNER, P., ROOS, G., JOHANSSON, H., LINDH, J. & DIGE, U. (1987). Non-Hodgkin's lymphoma. Multivariate analysis of prognostic factors including fraction of S-phase cells. *Acta. Oncol.*, **26**, 179.

MIYACHI, K., FRIZTLER, M.J. & TAN, E.M. (1978). Autoantibody to a nuclear antigen in proliferating cells. *J. Immunol.*, **121**, 2228.

ROSENBERG, S.A., BERARD, C.W., BROWN, B.W. & 5 others (1982). The non-Hodgkin's Lymphoma Classification Project: National Cancer Institute sponsored study on classification of non-Hodgkin's lymphomas. Summary and description of a working formulation for clinical usage. *Cancer*, **49**, 2112.

VAN DIERENDONCK, J.H., WIJSMAN, J.H., KEIJZER, R., CORNELIS, J.H., VAN DE VELDE & CORNELISSE, C.J. (1988). Cell-cycle related staining patterns of antiproliferating cell nuclear antigen monoclonal antibodies. *Am. J. Pathol.*, **138**, 1165.

WITZIG, T.E., GONCHOROFF, N.J., GREIPP, P.R. & 6 others (1989). Rapid S-phase determination of non-Hodgkin's lymphomas with the use of an immunofluorescence bromodeoxyuridine labeling index procedure. *Am. J. Clin. Pathol.*, **91**, 298.

WOODS, A.L., HALL, P.A., SHEPHARD, N.A., HANBY, A.M., ASEEM, N.H.W., LANE, D.P. & LEVISON, D.A. (1991). The assessment of proliferating cell nuclear antigen (PCNA) immunostaining in primary gastrointestinal lymphomas and its relationship to histological grade, S+G2+M phase fraction (flow cytometric analysis) and prognosis. *Histopathology*, **19**, 21.

YOUNG, G.A.R., HEDLEY, D.W., RUGG, C.A. & ILAND, H.J. (1987). The prognostic significance of proliferative activity in poor histology non-Hodgkin's lymphoma: A flow cytometry study using archival material. *Eur. J. Clin. Oncol.*, **23**, 1497.

YU, C.C.-W., HALL, P.A., FLETCHER, C.D.M., CAMPLEJOHN, R.S., ASEEM, N.H.W., LANE, D.P. & LEVISON, D.A. (1991). Haemangiopericytomas: the prognostic value of immunohistochemical staining with a monoclonal antibody to proliferating cell nuclear antigen (PCNA). *Histopathology*, **19**, 29.